# Genomic scan of selective sweeps in thin and fat tail sheep breeds for identifying of candidate regions associated with fat deposition

**DOI:** 10.1186/1471-2156-13-10

**Published:** 2012-02-26

**Authors:** Mohammad Hossein Moradi, Ardeshir Nejati-Javaremi, Mohammad Moradi-Shahrbabak, Ken G Dodds, John C McEwan

**Affiliations:** 1 Department of Animal Science-Excellent centre for improving sheep carcass quality and quantity, University of Tehran, PO Box 3158711167-4111, Karaj, Iran; 2 Centre for Reproduction and Genomics, AgResearch, Invermay, Mosgiel, New Zealand

## Abstract

**Background:**

Identification of genomic regions that have been targets of selection for phenotypic traits is one of the most important and challenging areas of research in animal genetics. However, currently there are relatively few genomic regions identified that have been subject to positive selection. In this study, a genome-wide scan using ~50,000 Single Nucleotide Polymorphisms (SNPs) was performed in an attempt to identify genomic regions associated with fat deposition in fat-tail breeds. This trait and its modification are very important in those countries grazing these breeds.

**Results:**

Two independent experiments using either Iranian or Ovine HapMap genotyping data contrasted thin and fat tail breeds. Population differentiation using F_ST _in Iranian thin and fat tail breeds revealed seven genomic regions. Almost all of these regions overlapped with QTLs that had previously been identified as affecting fat and carcass yield traits in beef and dairy cattle. Study of selection sweep signatures using F_ST _in thin and fat tail breeds sampled from the Ovine HapMap project confirmed three of these regions located on Chromosomes 5, 7 and X. We found increased homozygosity in these regions in favour of fat tail breeds on chromosome 5 and X and in favour of thin tail breeds on chromosome 7.

**Conclusions:**

In this study, we were able to identify three novel regions associated with fat deposition in thin and fat tail sheep breeds. Two of these were associated with an increase of homozygosity in the fat tail breeds which would be consistent with selection for mutations affecting fat tail size several thousand years after domestication.

## Background

The domestication of livestock represents a crucial step in human history. The rise of civilizations could not happen without domestication of plants and animals. Sheep (*Ovis aries*) is the first grazing animal known to have been domesticated [1]. Multiple mitochondrial lineages suggest that domestication occurred several times, as in other livestock species such as cattle, goat and pig [2]. Recognition of the origin of domestication is difficult by the fact that the first domestic animals were no different from their wild counterparts [3]. In spite of this, the archaeozoological evidence suggests that the domestication of sheep occurred during the Neolithic revolution approximately 9000 years ago [4] in a region in northern Iraq and nearby regions in Iran [5]. Since domestication, sheep have established in a wide geographical range due to their adaptability to poor nutrition diets, tolerance to extreme climatic conditions and their manageable size [6].

Fat tail breeds are an important class of sheep breeds that are first documented as being present 5000 years ago. The earliest depiction of a fat tail sheep is on an Uruk III stone vessel of 3000 BC and fat and thin tail sheep appear together on a mosaic standard from Ur dated around 2400 BC [3,7]. The fact that the fat tail breeds are now prevalent in the Fertile Crescent, where sheep were originally domesticated, while thin tail sheep breeds are predominant in peripheral areas [7] and that the wild ancestor of sheep is thin tail suggest that the first domesticated sheep were thin tail and fat tail was developed later. The evidence shows that sheep were being farmed throughout Europe 5000 years ago [8].

The fat tail is considered as an adaptive response of animals to a hazardous environment and is a valuable energy reserve for the animal during migration and winter. Until recently it had additional value to the herder because it was used to preserve cooked meat for longer periods of time and also as an energy reserve during times of drought and famine. Therefore the climatic variation as well as the associated requirements of humans led to artificial selection for higher fat tail weight across generations [9-11]. Nowadays in intensive and semi-intensive systems most of the advantages of a large fat tail have reduced their importance and therefore, a decrease in fat tail size is often desirable for producers and consumers. Fat deposition requires more energy than the deposition of lean tissue, animal fat has lost much of its market demand and monetary value and sheep producers have easy access to other forms of auxiliary feeding [12]. These breeds are commonly found in a wide range of countries in Asia especially the Middle East and North Africa [7]. To date, several investigations into the inheritance of fat tails have been undertaken [10,11,13-18], nevertheless the genes affecting fat deposition in fat tail breeds are still unknown.

The study of genes underlying phenotypic variation can be performed in two different ways, firstly from phenotype to genome, which is performed by LD based association mapping or by targeting particular candidate genes identified based on homology to known genes, and secondly from genome to phenotype, which involves the statistical evaluation of genomic data to identify likely targets of past selection using selective sweep analysis [19,20]. The elimination of standing variation in regions linked to a recently fixed beneficial mutation is known as a "selective sweep" and has recently been the focus of much theoretical and empirical attention [21-23].

In contrast to natural populations, domesticated species provide an exciting opportunity to understand how artificial selection promotes rapid phenotypic evolution [22]. With an hypothesis that different selection pressures operated in thin and fat tail breeds over the history of time and somehow the selection acts on a variant that is advantageous only in one breed, it is expected that the frequency of that variant may differ across populations to a greater extent than predicted for variants evolving neutrally in all populations [21]. Identifying of these genome regions, which have been subject to such selective sweeps could reveal the mutations which are responsible for the fat deposition in these breeds. The examination of variation in SNP allele frequencies between populations, which can be quantified by the statistic F_ST_, is a promising strategy for detecting signatures of selection [21,24].

To date a relatively small number of examples have successfully identified genomic regions subject to positive selection in different domestic animals [20,25-38]. The constraint to identifying selection signatures in sheep has been the limited density of markers. This limitation has recently been reduced with the availability of tens of thousands of single nucleotide polymorphisms (SNPs) using the Ovine SNP50k BeadChip (http://www.sheephapmap.org.) In this research two independent resources have been investigated to identify regions associated with fat deposition in these breeds. The first data set was comprised of one thin and one fat tail Iranian sheep breed while the second was comprised of several other fat and thin tail breeds selected from the Ovine HapMap project.

**Figure 1:**
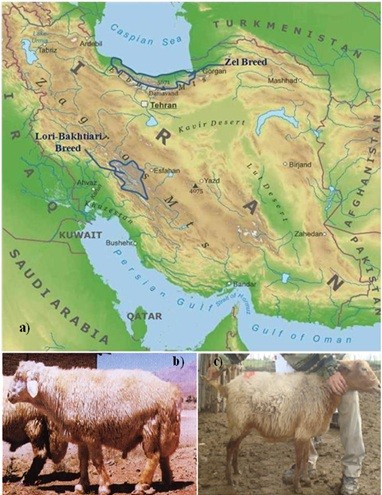
**Traditional distributions of the two Iranian breeds used in this study are shown in (a) with breed examples of Lori Bakhtiari (b) and Zel (c)**.

Sheep production constitutes the most important component of the Iranian livestock industry with a total of approximately 50 million head. Twenty seven breeds and ecotypes have been documented in Iran, the majority derived *in situ*. The Lori-Bakhtiari sheep breed is one of the most common indigenous breeds. It is well adapted to the hilly and mountainous Bakhtiari region stretched out to southern Zagros Mountains (Figure 1), with a population of more than 1.7 million. The animals are kept mostly in villages under semi-intensive systems. Relative to other Iranian fat tail breeds the Lori-Bakhtiari is a large breed, having the largest fat tail by girth and weight [39]. Sheep breeds vary in tail length and this breed, perhaps surprisingly, has a rather short tail length. Zel is the only thin-tail Iranian breed and it is present largely on the northern slopes of the Elburz mountain range near the Caspian Sea [40] representing around 3% of Iranian sheep.

The aim of this study was to find selective sweeps between the Iranian thin and fat tail breeds using dense SNP markers and to compare with those from similarly divergent breeds extracted from the Ovine HapMap dataset (http://www.sheephapmap.org.) Our work provides the first genome wide characterization of selective sweeps in thin and fat tail breeds. Each animal was genotyped with approximately 50,000 SNPs and a variety of selection sweep tests were utilized.

## Results

### Genotyping and data mining

**Figure 2:**
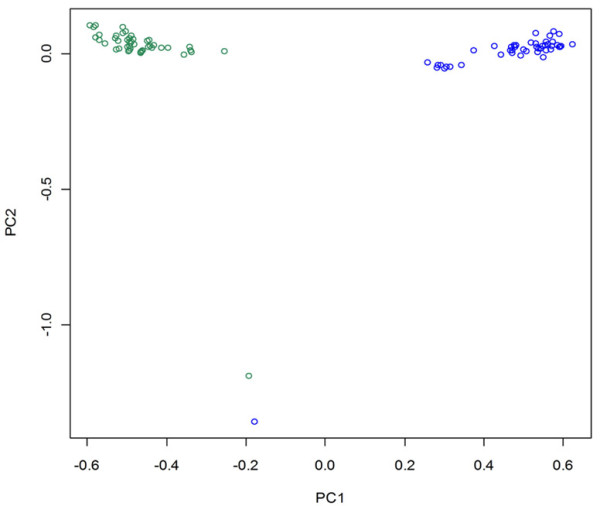
**Animals clustered on the basis of principal components analysis using individual genotypes**. Zel and Lori Bakhtiari breeds are shown by green and blue circles respectively.

A total of 94 animals consisting of 47 samples per breed were genotyped on the Illumina OvineSNP50K Beadchip assay in the Zel-Lori Bakhtiari data set. One animal in each breed had greater than 5% missing data and these were excluded from further analysis. The second data set used in this study was SNP genotyping data from similarly divergent breeds in the Ovine HapMap project (Table 1). After data cleaning (see methods) a total of 45,611 and 48,053 SNPs passed the filtering criteria in the Zel-Lori Bakhtiari and HapMap data sets respectively and were included in the final analyses. The overall average distances between 2 adjacent SNPs were relatively consistent among the chromosomes, being about 60 kb in the Zel-Lori Bakhtiari and 58 kb in the HapMap data sets. In all cases the locations used were obtained from OAR true chromosomes (ver.1.0, as at 5/2008) from CSIRO [41]. The principal component analysis (PCA) in Zel-Lori Bakhtiari data set, using the individual SNPs as the data, resulted in the first two principal components (PC1 and PC2) explaining 18.6% and 2.9% of the variance respectively. We found that PC1 separated out the two breeds from each other while one animal in each breed was separated from all other animals for PC2 (Figure 2). These two animals were excluded from further analysis. Finally, 45 animals (36 females and 9 males) per breed passed the data cleaning steps and 95% and 95.1% of all remaining SNPs were in Hardy-Weinberg equilibrium at the 5% level in Zel and Lori-Bakhtiari breeds respectively. For the SNPs analyzed in this study, the average MAF over all samples was 0.29 (SD = 0.13) in the Zel-Lori Bakhtiari data set and 0.30 (SD = 0.12) in the HapMap data set.

**Table 1:** Origin and sample size of different breeds included in this study

Data set	Breed	**Code**^**1**^	Tail status	Sampling area	Sample size
Zel-Lori Bakhtiari data set					
	Zel	ZEL	Thin tail	Iran	47
	Lori-Bakhtiari	LOR	Fat tail	Iran	47
Ovine HapMap data set					
	Deccani	DEC	Thin tail	India	23
	Scottish Black Face	SBF	Thin tail	United Kingdom	56
	Bunder Oberland Sheep	BOS	Thin tail	Switzerland	24
	Gulf Coast Native	GCN	Thin tail	North America	94
	Karakas	KAR	Fat tail	Turkey	18
	Norduz	NDZ	Fat tail	Turkey	20
	Afshari	AFS	Fat tail	Iran	37

^1 ^Code is the 3-letter code for each breed.

### Genomic distribution of F_ST _in Zel-Lori Bakhtiari data set

**Figure 3:**
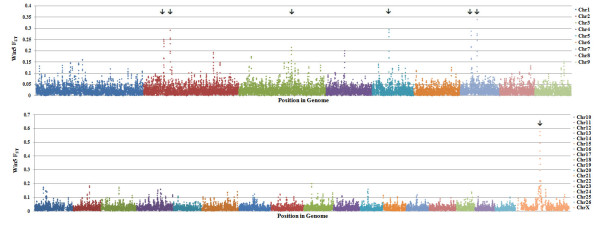
**Distribution of windowed F_ST _values for Zel versus Lori-Bakhtiari breeds by chromosome**. SNP position in the genome is shown on the X-axis, and windowed F_ST _is plotted on the Y-axis. Regions with arrows above had windowed F_ST _value > 0.20 and were later examined for further analysis.

The plot of windowed F_ST _against location is shown in Figure 3. The average of differentiation between Zel and Lori Bakhtiari breeds was 0.024 (SD = 0.035). As shown in this figure, in several instances outlier SNPs tended to cluster to similar regions. Specifically, we found evidence of selection in seven regions with windowed F_ST _value > 0.20 on chromosomes 2 (between 55,861-56,300 kb), 2 (between 73,631-73,784 kb), 3 (between 146,615-146,676 kb), 5 (between 47,149-47,263 kb), 7 (between 30,512-30,585 kb), 7 (between 46,642-46,843 kb) and X (between 58,621-61,452 kb).

The average F_ST _for autosomal and X-linked SNPs was significantly different (0.024 and 0.035, respectively; t test, t = 6.2, P < 10^-10^). A higher average F_ST _for X-chromosome SNPs could occur because of its smaller effective population size compared with that of the autosomes.

Another genetic distance measurement including unbiased estimates of F_ST _as described by Weir and Cockerham [42] was also calculated. Because the results were highly correlated (r = 0.995) with the above results, so have not been presented.

### Study of Bovine genes and published QTLs in regions showing evidence of selection

Seven regions showing the largest signals of selection in Zel-Lori Bakhtiari data set were chosen for further analysis. As the current annotation of the sheep genome is not as comprehensive as cattle, the regions of interest in *O. aries *were compared to the corresponding areas in *B. taurus*. Dot plots for corresponding areas of Ovine and Bovine genomes showed strong co-linear relationships between the two considered sequences in all regions and rearrangements were not observed (Additional file 1: Figure S1).

A summary of orthologous area in both species and published bovine genes and QTLs is presented in table 2. Orthologus genes in the bovine genome were identified using the BLAT genome search with UCSC Genome Browser [43].

**Table 2:** Bovine genes and published QTLs in regions showing evidence of selection in Zel vs Lori Bakhtiari data set

Chr-Region	Location on Ovine genome	Location on Bovine genome	Gene*	RefSeq Number	QTL	QTL Reference
2-1	2:55859169-56302905	8:62601056-63034390	*NPR2*	NM_174126.2	Fat Depth	[44]
			*SPAG8*	NM_001102005.1	Carcass weight	[45]
			*HINT2*	NM_174340.2	Body weight (birth)	[45]
			*TMEM8B*	NM_001103335.1		
2-2	2:73628609-73861345	8:45226663-45415081	*---*		Fat Depth	[44]
					Fat thickness	[44]
					Carcass weight	[45]
					Body weight (birth and mature)	[45]
3	3:146615284-146676235	5:33981017-34041987	*CACNB3*	NM_174509.3	Meat	[46]
					Tenderness	
			*DDX23*	NM_001102202.1	Hot Carcass Weight	[47]
					Birth Weight	[48,49]
5	5:47146900-47332222	7:44936435-45113988	*PPP2CA*	NM_181031.2	Fat thickness	[48]
						
			*SKP1*	NM_001034781.1		
			*TCF7*	NM_001099186.1		
7-1	7:30510065-30587784	10:29363930-29441529	*----*		Milk fat yield	[50]
					Carcass weight	[48]
					Hot Carcass Weight	[48]
					Body weight (birth)	[45]
7-2	7:46639859-46910155	10:45312778-45581699	*PTGDR*	NM_001098034.1	Milk fat yield	[50]
					Hot Carcass Weight	[48]
					Body depth	[51]
X	X:59192476-60151772	X:51751347-52712765	*---*			

* Obtained by orthology with the Bovine genome

Online databases of published QTL in beef and dairy cattle, show that the regions identified here were previously been found to be in regions harboring QTL affecting fat and also carcass yield traits (Table 2). For example, the regions on chromosomes 2 overlapped with QTLs previously suggested as being related with fat depth, and also chromosomes 2 (second region) and 5 with fat thickness and both regions on chromosomes 7 with milk fat yield.

### Genomic distribution of F_ST _in Ovine HapMap data set and comparison with Zel-Lori Bakhtiari data set results

**Figure 4:**
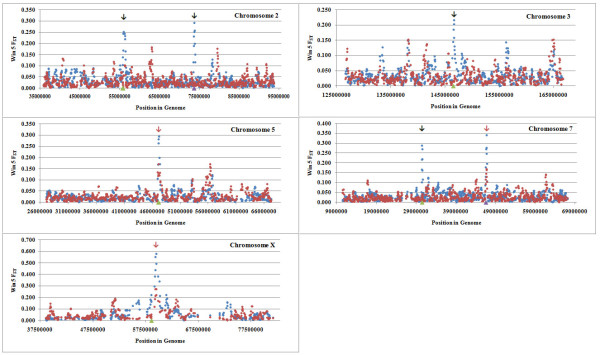
**Distribution of windowed F_ST _values for Zel-Lori Bakhtiari and HapMap data sets for candidate regions by chromosome**. SNP position in the genome is shown on the X-axis, and windowed FST is plotted on the Y-axis. All arrows show regions with largest allele differentiation in Zel-Lori Bakhtiari data set and the red arrows are highlighting the peaks that confirmed by HapMap dataset. Zel-Lori Bakhtiari and HapMap data sets are shown by blue and red circles respectively.

An independent resource comprised of similarly divergent breeds for thin and fat tail breeds in the Ovine HapMap project were selected to determine whether the regions with large allele differentiation in Zel-Lori Bakhtiari data set could be confirmed. The pattern of F_ST _across the genome was also calculated in the Ovine HapMap data set (fat and thin tail breeds were pooled) and values were averaged in a sliding window. The windowed F_ST _was then plotted against location in the genome. The windowed F_ST _values, at each common SNP, were correlated in the two data sets with r = 0.413, N = 45,238 and the average of differentiation between thin and fat tail breeds in the Ovine HapMap data set was 0.027 (SD = 0.038). The sliding window average F_ST _revealed good agreement between both data sets for regions on chromosomes 5, 7 (second region) and X (Figure 4).

Calculation of empirical p-values for windowed F_ST _values in each data set (Table 3) shows that the 3 regions with the largest differentiation in Zel-Lori Bakhtiari data set are also significant in the Ovine HapMap data set (P < 0.01). On the other hand, combining p-values from independent tests of significance in Zel-Lori Bakhtiari and Ovine Hapmap data sets using Fisher's combined test revealed the first region on chromosome 2 (P < 0.0001) in addition to these same significant regions.

**Table 3:** The table of significant test for Zel-Lori Bakhtiri and ovine HapMap data set.

SNP_Name	Chr	Coordinate	Zel-Lori Bakhtiri data set		HapMap data set		Fisher's combined
				
			**windowed F**_**ST**_	P-value	**windowed F**_**ST**_	P-value	P-value
OAR2_55861669.1	2	55861669	0.24	0.00051	0.06	0.08192	0.0004593
s63030.1	2	55922894	0.25	0.00042	0.06	0.07696	0.0003646
s20468.1	2	56248983	0.24	0.00048	0.10	0.01297	0.0000814*
s58048.1	2	56288982	0.23	0.00053	0.10	0.01303	0.0000886*
OAR2_56300406.1	2	56300406	0.22	0.00064	0.10	0.01441	0.0001157
s42805.1	2	73631109	0.23	0.00055	0.02	0.52322	0.0026316
OAR2_73687773.1	2	73687773	0.25	0.00044	0.02	0.46291	0.0019329
OAR2_73744625.1	2	73744625	0.29	0.00024	0.02	0.42839	0.0010538
OAR2_73784818.1	2	73784818	0.26	0.00040	0.03	0.38720	0.0014986
s35724.1	3	146673736	0.22	0.00066	0.01	0.71086	0.0040613
s55322.1	5	47149400	0.26	0.00037	0.13	0.00505	0.0000267*
OAR5_47175489.1	5	47175489	0.28	0.00029	0.17	0.00140	0.0000063*
OAR5_47263230.1	5	47263230	0.29	0.00022	0.12	0.00669	0.0000212*
OAR7_30512565.1	7	30512565	0.22	0.00068	0.02	0.45742	0.0028276
s54240.1	7	30525058	0.29	0.00026	0.03	0.25759	0.0007199
s67498.1	7	30572812	0.27	0.00035	0.04	0.19337	0.0007205
OAR7_30585285.1	7	30585285	0.22	0.00062	0.04	0.17072	0.0010675
OAR7_46642359.1	7	46642359	0.27	0.00033	0.15	0.00282	0.0000138*
OAR7_46765080.1	7	46765080	0.34	0.00015	0.16	0.00177	0.0000044*
OAR7_46818598.1	7	46818598	0.28	0.00031	0.17	0.00161	0.0000077*
OAR7_46843356.1	7	46843356	0.25	0.00046	0.13	0.00476	0.0000308*
OARX_58621412.1	X	58621412	0.22	0.00057	0.05	0.09020	0.0005604
OARX_59194976.1	X	59194976	0.30	0.00020	0.12	0.00647	0.0000186*
OARX_59257971.1	X	59257971	0.38	0.00013	0.19	0.00088	0.0000020*
OARX_59327581.1	X	59327581	0.44	0.00009	0.21	0.00048	0.0000008*
OARX_59383635.1	X	59383635	0.55	0.00004	0.27	0.00019	0.0000002*
OARX_59571364.1	X	59571364	0.58	0.00002	0.27	0.00017	0.0000001*
OARX_59578440.1	X	59578440	0.49	0.00007	0.22	0.00038	0.0000005*
OARX_59912586.1	X	59912586	0.38	0.00011	0.17	0.00144	0.0000026*
s38079.1	X	60149273	0.34	0.00018	0.16	0.00179	0.0000050*
OARX_60238540.1	X	60238540	0.21	0.00070	0.07	0.03783	0.0003069
s31917.1	X	61452816	0.22	0.00059	0.04	0.19923	0.0011873

Position, windowed F_ST_, empirical P-values and Fisher's combined P-values of observed peaks in Zel-Lori Bakhtiri data set (windowed F_ST _> 0.20) and comparison to independent experiment using Ovine HapMap data

* Significant *P*-Value < 0.0001

The Weir and Cockerham method of estimating F_ST _[42] was also performed for the ovine HapMap data and a high correlation (r = 0.992) was observed. Previous reports [e.g. [24,52]] also indicated these methods led to similar results; therefore, a strong correlation between these two measures is not surprising.

### Study of median homozygosity in candidate regions

**Figure 5:**
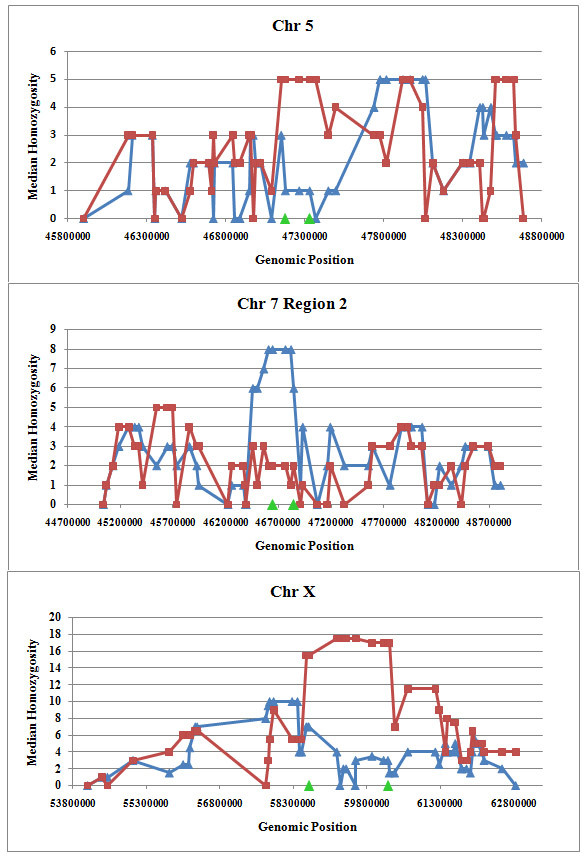
**Median homozygosity run length (n = 50 SNPs) surrounding candidate regions in Zel (blue triangular) and Lori Bakhtiari (red square) breeds**. Regions with largest F_ST _(> 0.20) value are shown on the X-axis.

Median run of homozygosity (Figure 5) was increased at the candidate regions on chromosome 5 and X for Lori Bakhtiari (fat tail) and at the candidate region on chromosome 7 for Zel (thin tail). The largest differences of median homozygosity were located on chromosome X and this was present for a longer distance as well, whereas these statistics were lower on Chromosome 5. A study of median homozygosity in the HapMap data set for thin and fat tail breeds (data not shown) revealed similar results.

### Estimation of effective population size and haplotype age

The average extent of LD in the genome was used to estimate the effective population size at various times in the past. The estimated effective population size show a persistent decline from 2000 down to 20 generations ago, declining from 4900 (both breeds) to 840 in Zel and 532 in Lori Bakhtiari.

The graph of effective population sizes suggests a distinctive time point when the breeds separated ~1,100 generations ago (Additional file 2: Figure S2). Assuming an average generation interval in sheep of around 5 years or 5500 before present, this is congruent with the first archaeological evidence for the fat tail sheep breeds (~5,000 years ago).

The average effective population size over the period was calculated using reciprocals as described by Falconer [53]. Using this average estimated effective population size and based on the current frequency of sweeps in our regions of interest [54], the age of sweeps under the assumption of selection neutrality and genetic drift has been inferred for chromosomes 5, 7 and X to be approximately 7100, 9600, and 6900 generations before present respectively.

These estimated ages should not be taken to imply a burst of selection at a particular time; however, these ages might represent areas in the parameter space in which we have good power.

## Discussion

Approximately 25% of the world sheep population comprises fat tail breeds that are grazed in a wide range of countries especially in Asia and northern parts of Africa. In these typically arid countries sheep breeding has an important place in the local economy and as a source of protein. Due to the overt and easily defined nature of a fat tail, the gene variants affecting the phenotypic expression of this trait are a topic of both theoretical and economic curiosity. The former from the perspective of the impact and nature of domestication and the latter because the trait is now markedly less important commercially due to improved forage availability and decreased price for the product.

In this research we have developed an initial selection map for the fat tail trait in sheep. The objective was to localize genomic regions potentially affecting the trait. This was undertaken by comparing fat and thin tail breeds using the newly available 50K SNPChip. The chip has already proven its ability to map causal mutations for traits showing Mendelian inheritance such as yellow fat [55] and microphthalmia [56].

It has been previously shown in cattle, that domestication, subsequent breed formation and artificial selection, leave detectable signatures of selection in several regions of the genome [36]. Given the similarities in domestication time and selection between cattle and sheep, it is likely that the ovine genome contains similar signatures.

The Iranian breeds were chosen for this study, because they originated both near the centre of domestication [5] and near the first recorded archaeological evidence of fat tail sheep [3,7]. It was felt this geographic proximity would reduce false positives due to bottlenecks and selection pressure due to alternative factors such as climate, disease and pasture types. However, we expected that some signals would still be spurious so we then independently validated the results using a different set of breeds obtained from the Ovine HapMap project. The latter project did not formally phenotype the individuals concerned so these breeds were classified by us solely on publicly available breed descriptions. It is important to note that the selection sweep approach is one of the few viable approaches for investigating the genomics of this trait given the poor level of historical recording and DNA sampling in the relevant breeds. It is also one of the most cost effective and powerful techniques albeit as previously stated also subject to potential false positive signals.

Several statistical approaches have been devised to detect evidence of selective sweeps. These different statistics detect different aspects of the pattern of variation left by selection of a beneficial mutation. Generally, the power of the tests also depends on the strength, duration and age of the selective sweep [21]. Unlike F_ST_, tests based on linkage disequilibrium like iHS [57] and XP-EHH [58] which are used primarily on human SNP data, where there are now millions of SNPs available, are dependent on SNP spacing and frequency as they are multi-marker tests. The 50,000 SNPs available for sheep may not provide enough information for these tests; it has been suggested in cattle that 150,000 evenly spaced SNPs would be required to study selective signatures in all parts of the bovine genome [29]. Additionally, the power of these methods also depends on the ancestral allele information which is still available for only a portion of the SNPs on the ovine chip.

However, it should be considered that robust inference of recent positive selection using F_ST _is complicated by the fact that the distribution of genetic variation effect due to selection can be difficult to distinguish from that which arises after certain demographic events. Some previous studies that have attempted to identify signatures of selection based on patterns of population differentiation have used simulations to obtain the expected distribution of F_ST _under the assumption of no selection [24,33,34]. However, the simulated distribution of F_ST _is strongly dependent on its underlying assumptions about population demographic history, which is rarely known with any degree of certainty [24,37]. Despite this problem, examining several thousand loci using newly available SNP chips provides an exciting opportunity to distinguish between the effects of population structure, positive selection and ascertainment bias. Demographic events and ascertainment bias would be expected to alter patterns of F_ST _across the whole genome in a similar way, while selection events would be expected to alter F_ST _values only in selected and nearby loci [29-34].

In general, we found a low differentiation between populations, with a mean of 0.024 (SD = 0.035) for the Zel-Lori Bakhtiari data set and 0.027 ± 0.038 for the HapMap data set. This agrees with Kijas et al. [6] who, using 23 domestic breeds and two wild sheep species, revealed that sheep breeds showed generally low differentiation which is consistent with their short evolutionary history.

The general pattern of the signals across chromosomes was different in the HapMap data set and there were differences in the locations of the major peaks (data not shown). However, Barendse et al. [29] observed similar results when comparing Bovine Australian and HapMap data. Their windowed F_ST _values between two data sets were correlated with r = 0.346, whereas this correlation in our data sets was r = 0.413. In general this correlation with different breeds may suggest that each study had identified signals of divergence particular to the genetic history of those breeds, and only some of which may be due to selection (as described by Barendse et al. [29]). This correlation for our candidate regions was improved to r = 0.963.

We found few genes in the regions of interest and no particular candidate genes related to fat deposition were identified. However, the current annotation of the cattle genome is not as comprehensive as in humans, so these areas cannot be dismissed as not containing any genes or regulatory elements. It has been observed that while some proposed candidates for selection have strong support in the form of a functional mutation with an identified phenotypic effect, often the functional target is unknown [21]. As the cattle genome becomes more comprehensively annotated in these regions likely targets of selection may be identified.

A variety of alternative comparisons, with the various thin and fat tail breeds using the combined HapMap and Zel-Lori Bakhtiari data set, can be examined in addition to the comparisons presented here. In a comparison, when we compared some fat tail breeds together (Afshari and Lori Bakhtiari breeds), we found the F_ST _peak in the same location on chromosome 2 region 1 which was significant based on Fisher's combined test. The orthologous area for this region (using Blat) on the cattle genome would be around 8:62,600,000-63,000,000 (Table 2). Several bovine studies have also detected signatures of selection in this region. MacEachern et al. [34] compared differences in allelic frequencies of Australian Angus and Holstein cattle at 7,500 SNPs. They reported a region with large differences among breeds at 61,300,000 to 62,500,000 bp on BTA8. Stella et al. [37] observed a significantly large signal of selection in the same location for the Holstein and Jersey breeds. Although neither study has reported any particular candidate gene for this observation, previously identified QTLs (Table 2) could suggest a candidate region for body composition and carcass traits in this chromosomal region.

Using median homozygosity plot in these regions, we identified that homozygosity has been increased on chromosome 5 and X in favour of fat tail breeds. This is consistent with a recessive mode of inheritance. Therefore future studies should track both the phenotype of this trait and the genotype status in these regions in F_2 _crosses to provide independent and causal evidence that these regions do in fact affect the fat tail trait and to verify its mode of inheritance.

Given that one of the most differentiated regions was located on chromosome X, this suggests sex linked differences could be present. In several unpublished experiments when a research institute in central Iran crossed wild sheep (male) with a couple of fat tail ewes, according to the reports all resulting lambs were thin tail. This suggests dominance of the thin tail phenotype and does not support a simple single gene located on sex chromosome alone. Similarly, when two European thin tail breeds were crossed with Iranian fat tail ewes, all of the more than 350 crossbred lambs of both sexes subsequently born were thin tail and no fat tail lambs were observed. This supports the previous observations and again does not support sex linked effects, unless some epistatic effects exist or any relevant single gene located on chromosome X should be located in a pseudo-autosomal regions.

In general, there is little published information related to tail fatness in crosses between native fat and thin tail sheep breeds and also the relatively small number of animals used in these studies makes it difficult to obtain reliable estimates pertaining to this question [10,11,13-18]. However, in several experiments measurements suggest crosses were intermediate between fat and thin tail sheep [11,17]. This conflicts with the unpublished Iranian results and does raise the possibility that the results observed may depend on the fat and thin tail breeds used in the cross. In our opinion, more detail and larger scale experiments are needed to confirm the results on growth and carcass characteristics of crossbred lambs of different sexes. If such studies are undertaken the nature of inheritance on the X chromosome can be rather difficult to elucidate, especially if imprinting is also suspected [59].

A result which is irrelevant to the inheritance of the trait, but provides an insight into a possible mechanism of fat deposition in this organ, are the results of Gökdal et al. [10] who examined the effects of docking in fat tail breeds. The carcasses of the docked group contained more kidney, pelvic and internal fat than the intact lambs as well as a higher percentage of subcutaneous and intramuscular fat. The weights of the different carcass cuts of the docked lambs were also heavier that those of the intact group. However, there was little change in overall carcass composition suggesting that the genes affecting the fat tail phenotype are associated with the localization of fat stores to a regional depot rather than control of the overall level of fat deposition. This observation also may provide support to the suggestion that this trait is a result of human mediated selection as it is difficult to postulate any natural selection criterion favoring deposition in this area.

Finally, a search for comparable depots in other species especially grazing mammals identified Bacterian humps in camels and humps in bos indicus cattle breeds as possible analogous features that have been selected for in animals grazing in arid and tropical regions subjected to wide fluctuations in food supply. The closest equivalent structure present in all mammals is possibly the mammary gland with its associated mammary fad pad. The current work, while not able to answer these hypotheses about the origin of the fat tail and analogous structures in other species directly, does offer hope that these questions will soon be able to be answered by further experimentation.

## Conclusions

In this paper, we present the first genome wide characterization of selective sweeps in thin and fat tail breeds with the aim of identifying regions associated with fat deposition. This trait is one of the most challenging areas of research in most countries grazing fat tail breeds. The analysis was performed on two independent resources using either Iranian or Ovine HapMap genotyping data for thin and fat tail breeds. Population differentiation using F_ST _in Iranian thin and fat tail breeds revealed seven genomic regions. Almost all of these regions overlapped with QTLs that had previously been identified as affecting fat and carcass yield traits in beef and dairy cattle. Study of selection sweep signatures using F_ST _in thin and fat tail breeds sampled from the Ovine HapMap project confirmed three of these regions located on chromosomes 5, 7 and X. Using a median run of homozygosity plot in these regions, we identified that homozygosity has been increased on chromosome 5 and X in favour of fat tail breeds and on chromosome 7 in favour of thin tail breeds. Those associated with an increase of homozygosity in the fat tail breed would be consistent with selection for mutations affecting fat tail size several thousand years after domestication.

## Methods

### Animal samples

In this research two independent data sets were used to compare frequencies of SNP alleles in thin and fat tail breeds.

### Zel-Lori Bakhtiari data set

Blood samples were collected from flocks which have recently utilized the registration and recording system of the ABCI (Animal Breeding Centre of Iran). Two factors were considered for selecting the samples: selection of unrelated animals and sampling those that spanned the diversity of the breed. When sampling in pedigreed animals we ensured that they had no common grandparents and in non pedigreed animals we typically selected 4-5 animals from each flock each from different age classes. All animals had the following phenotypic records measured: herd no., location, GPS coordinates, sex, horn status, parity, approximate age, wool and skin colour, fat tail dimensions, height and weight. Animal sampling for the Zel breed was performed in the northern province of Mazandaran, south of the Caspian Sea and for the Lori-Bakhtiari breed in the Chaharmahal and Bakhtiari provinces, located in the south western part of Iran close to the Zagros Mountain ranges on farms distributed across the traditional rearing area of each breed (Figure 1). In general, more than 100 blood samples per each breed were collected and finally 47 samples were selected to be genotyped in each breed.

### Ovine HapMap data set

The second data set used in this study were genotypes of similarly divergent breeds from the Ovine HapMap project of the ISGC (International Sheep Genomics Consortium) and are available to the public at http://www.sheephapmap.org. There are many thin tail breeds in the HapMap project, so four breeds from geographically diverse locations and similar tail shape to Zel breed were selected. Since fat tail breeds in the HapMap project were mostly from a relatively narrow geographic region and their number was limited, three breeds with similar histories and fat tail shape to Lori Bakhtiari breed was selected (Table 1).

### Genotyping and data quality control

The Ovine HapMap project genotype data were downloaded from http://www.sheephapmap.org/download.php. Genomic DNA in the Zel-Lori Bakhtiari data set was extracted from whole blood by applying a modified salting out protocol [60] and DNA samples diluted to 50 ng/ul for genotyping. A total of 94 animals consisting of 47 samples per breed were genotyped on the Illumina OvineSNP50K Beadchip assay at the Centre for Reproduction and Genomics (CRG), Invermay, New Zealand using standard procedures (http://www.illumina.com) The same set of 49,018 SNPs randomly distributed across the genome which passed the HapMap criteria [61] were used in both data sets.

To ensure the overall quality of samples and a consistent set of genotypes, quality control filters were applied to the initial data. All samples with more than 5% missing data were excluded from further analysis. The rationale for this is that DNA which is not of high quality will be more likely to have more missing data and also have incorrect genotype calls [29]. Then for each SNP, minor allele frequency (MAF) and percentage of calls (how many sheep the marker worked for) was calculated. The SNPs that had a call rate less than 95% and a MAF (over all animals) less than 2% were discarded [62]. For the remaining SNPs outlier departure from Hardy-Weinberg equilibrium (p < 10^-6^) over all animals of a breed were used for identifying genotyping errors. Although departure from HWE might result from selection, it is most likely that technical problems explain this result and they were therefore excluded from subsequent analyses [63]. The Bonferroni correction (β = α/n) was used to address the problem of multiple comparisons [64]. The number of tests was taken to be the number of SNPs (n = 50,000) giving β = 10^-6 ^corresponding to α = 0.05 experiment-wise error. A principal component analysis (PCA) was performed using the prcomp function in the R package and the samples which were located outside of their expected breed cluster were excluded from further analysis. Finally markers were filtered to exclude loci assigned to unmapped contigs. The HapMap genotyping data had already passed the quality control process and only MAF and call rate were considered (Additional file 3: Table S3 and Additional file 4: Table S4).

### Analysis

#### Estimates of Wright's F_ST _and Weir & Cockerham's Theta

To determine a genome wide pattern of positive selection, and to compare this with the Ovine HapMap project samples, the basic form of Wright's fixation index (F_ST_), which measures the degree of genetic differentiation between subpopulations, based on genetic polymorphism data, was calculated as described by MacEachern et al. [34]:

$$F_{ST}=\frac{H_{T}-H_{S}}{H_{T}}$$

Where H_T _denotes the expected heterozygosities for overall total populations,

$$H_{T}=1-\sum \left(\overset{-2}{p}-\overset{-2}{q}\right)$$

And H_S _denotes the expected heterozygosities in subpopulations,

$$H_{S}=\frac{\sum_{i=1}^{n}H_{\exp i}\times n_{i}}{N_{Total}}$$

In the above formula, $\bar{p}$ and $\bar{q}$ stand for the frequency of allele A_1 _and A_2 _over the total population and H_expi _and n_i _denote expected heterozygosity and sample size in subpopulation i.

All F_ST _values in this study are for a single locus. The value of F_ST _can theoretically range from 0 (no differentiation) to 1 (complete differentiation, in which populations are fixed for different alleles). Signatures of positive selection can be recognized when adjacent SNPs all show high F_ST _[24], due to the hitch-hiking effect [65], implying divergent selection between breeds.

Smoothing, where a moving average of a certain number of markers is taken, is a method of looking for regions where selection is apparent over multiple markers, rather than one-off high values. The optimal size of the window depends on how long ago the selection sweep occurred, as LD breaks down with time. An arbitrary window of 5 markers (~300 Kbp) was chosen as it appeared to provide the better signal. Smoothing raises the issue of an appropriate adjustment for multiple testing, but we use it only for graphic illustration, with the singular SNP values used when looking at specific regions. The windowed F_ST _values were plotted against genome location.

One of the main problems with Wright's measure of F_ST _is that it does not account for sampling error. This was corrected for by Weir and Cockerham [42] who developed the unbiased estimator. We used this method in both data sets. As originally defined [66], the range of F_ST _is between 0 and 1. However, it is possible for the above unbiased estimate of F_ST _to assume negative values as well [24].

By ranking the sliding window F_ST _in the each data set, the empirical P-value was calculated for each SNP as the proportion of SNPs with a sliding window F_ST _value at least as extreme as the value for that SNP.

We applied Fisher's [67] method to combine the results from the k = 2 independent resources used, using the following statistic which follows a chi-square distribution with 2k degrees of freedom, under the null hypothesis:

$$X^{2}=-2\overset{K}{\underset{i=1}{\sum }}\log_{e}\left(p_{i}\right)$$

Where, p_i _is the p-value for the i^th ^hypothesis test. The significance level considered for each experiment was 10^-2 ^and the chosen level for combined experiment was 10^-4^.

#### Study of genes and QTLs in our candidate regions

It is useful to compare regions of interest in *O. aries *to the corresponding areas in *B. taurus*, as the taurine genome is better annotated. Before comparisons of Ovine and Bovine sequences, dot plots were created using Gepard [68], with Ovine (OAR true chromosomes v 1.0) sequences obtained from Ovine Gbrowse [69], and Bovine (btau4) sequences obtained from the UCSC Genome Browser [43]. A dot plot is a graphical method for comparing two sequences, and identifies regions of similarity between them by organizing one sequence on the x-axis, and another one on the y-axis of a plot [70]. When the residues of both sequences match at the same location, a dot is drawn. The straightness of the line is indicative of the relationship between the two sequences.

In order to discover if any of the regions implicated by the whole genome analysis contained genes of interest we used OAR true chromosomes (ver.1.0, as at 5/2008) from CSIRO [41]. After obtaining the Ovine sequence of our candidate regions using this browser, the genes in cow genome were identified by BLAT search using the UCSC Genome Browser on Cow Oct. 2007 (Baylor 4.0/bosTau4) Assembly. BLAT on DNA is designed to quickly find sequences of 95% and greater similarity of length 25 bases or more [43]. Gene function was determined using Online Mendelian Inheritance in Man (OMIM) at http://www.ncbi.nlm.nih.gov/omim/ and Uniprot at http://biogps.gnf.org/.

We also explored two QTL databases available online http://genomes.sapac.edu.au/bovineqtl/index.html, http://www.animalgenome.org/QTLdb/cattle.html to identify any overlapping of the candidate regions with published QTL in dairy and beef cattle.

#### Median run of homozygosity

One method for looking for selection between breeds and also finding the breed that has been under selection is a comparison of homozygosity in a region. When a causal mutation is selected for in one breed and not the other, one breed would show high homozygosity in a genomic interval while the other does not. For each SNP in each animal, the length of a run of homozygosity (number of consecutive homozygous SNPs including the one being considered) was calculated (this would be zero if the SNP being considered was heterozygous). For each marker the median length, over the breed, of the run of homozygosity was calculated and plotted against genomic position in the candidate regions (25 SNPs on each side) for each breed. This is similar to looking at LD in those areas where a run of homozygosity (and therefore a high median homozygosity) indicates selection. Median homozygosity on chromosome X was calculated using only females (36 animals per each breed).

#### Estimation of Ne and haplotype age

A pair of haplotypes was estimated for each animal in the sample using fastPHASE Version 1.2 [71]. The estimated pairwise haplotype frequencies were used to calculate the squared correlation coefficient between the 2 loci (r^2^) following Hill and Robertson [72] as:

$$r^{2}=\frac{{\left(freq\left(A1-B1\right)\times freq\left(A2-B2\right)\;-\;freq\left(A1-B2\right)\times freq\left(A2-B1\right)\right)}^{2}}{freq\left(A1\right)\times freq\left(A2\right)\times freq\left(B1\right)\times freq\left(B2\right)}$$

Where for example freq (A1-B1) is the frequency of haplotype marker 1 allele 1, marker 2 allele 1, freq (A1) is the frequency of marker 1 allele 1, and freq (B1) is the frequency of marker 2 allele 1 [27].

The effective population size was then estimated using the approximate expectation of *r*^2 ^as:

$$E\left(r^{2}\right)=\frac{1}{1+4N_{t}c}$$

Where *N_t _*is the effective population size 1/(2*c) *generations in the past, *E*(*r*^2^) is estimated by the average of *r*^2 ^values for all pairs of SNPs, and *c *is the median of distances in Morgans [27,73-75].

To obtain a crude estimate of the ages of sweeps, we used the age estimation based on current frequency as -4*N *[*p*(log *p*)/(1 - *p*)], where N is effective population size and p is the current frequency of the derived allele (selected sweep) [53].

## Authors' contributions

MHM planned, performed the analyses and drafted the manuscript, ANJ contributed to the Iranian data set collection and supervised the analysis, MMS coordinated the study and sample collection, KGD provided statistical and analysis support and JCM supervised the analysis and provided the Ovine HapMap Data set. All authors have contributed to the editing of the article, and approved the final manuscript.

## Supplementary Material

Additional file 1 **Figure S1**: Dot plots comparing ovine sequences (y axis), and their corresponding area on bovine genome (x axis) for different regions. Click here for file

Additional file 2 **Figure S2**: Change in effective population size across generations as estimated from linkage disequilibrium data. Click here for file

Additional file 3 **Table S3**: Summary table for data cleaning in the Zel-Lori Bakhtiari data set. Click here for file

Additional file 4 **Table S4**: Summary table for data cleaning in the Ovine HapMap data set. Click here for file
