# Supplementary material for: Genomic scan of selective sweeps in thin and fat tail sheep breeds for identifying of candidate regions associated with fat deposition
Source: BMC Genet. 2012 Feb 26;13:10. doi: 10.1186/1471-2156-13-10 (PMC3351017; doi:10.1186/1471-2156-13-10)
Supplement: Additional file 1 — Figure S1: Dot plots comparing ovine sequences (y axis), and their corresponding area on bovine genome (x axis) for different regions. [file 1471-2156-13-10-S1.PDF]

Additional file 1: Dot plots comparing ovine sequences (y axis), and their corresponding area on bovine genome (x axis) for different regions:

Chr. 2-1

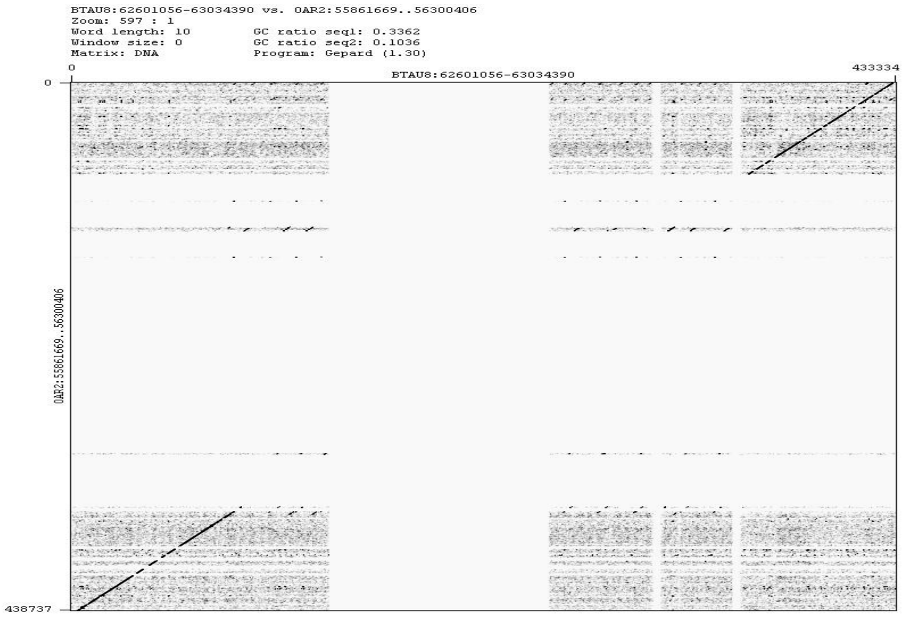

Chr. 2-2

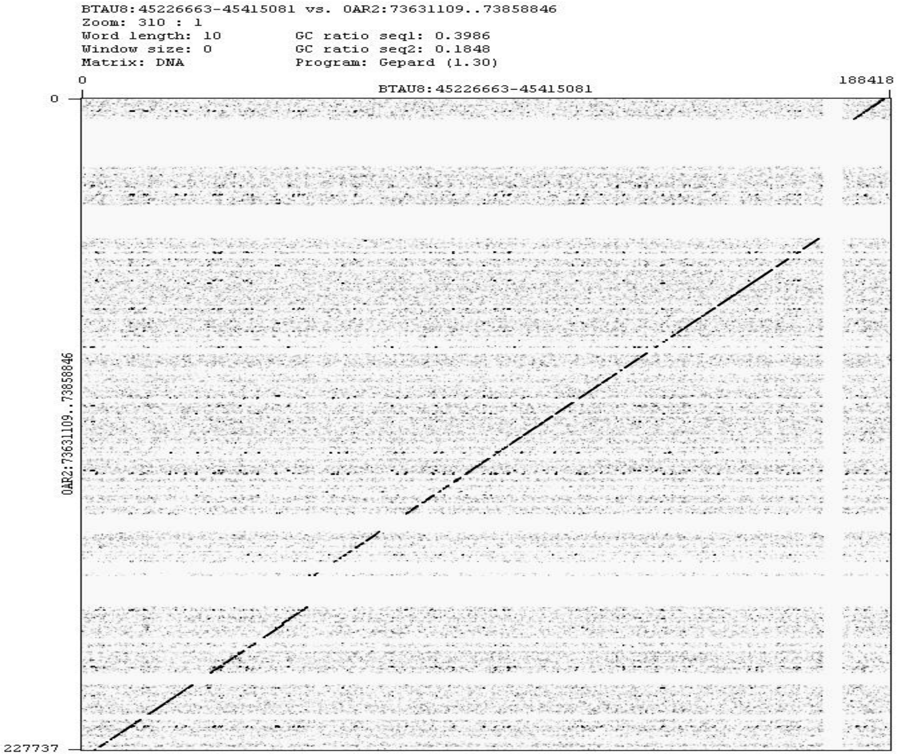

Chr. 3

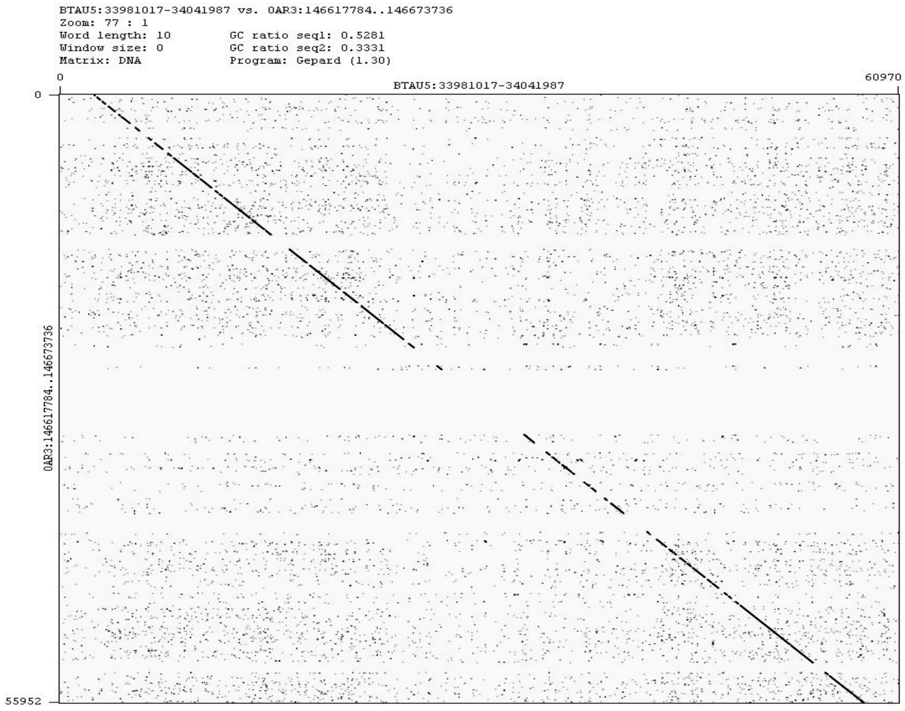
BTAU5:33981017-34041987 vs. OAR3:146617784..146673736
Zoom: 77 : 1
Word length: 10
Window size: 0
Matrix: DNA
GC ratio seq1: 0.5281
GC ratio seq2: 0.3331
Program: Gepard (1.30)
0
BTAU5:33981017-34041987
60970
0
OAR3:146617784..146673736
55952

Chr. 5

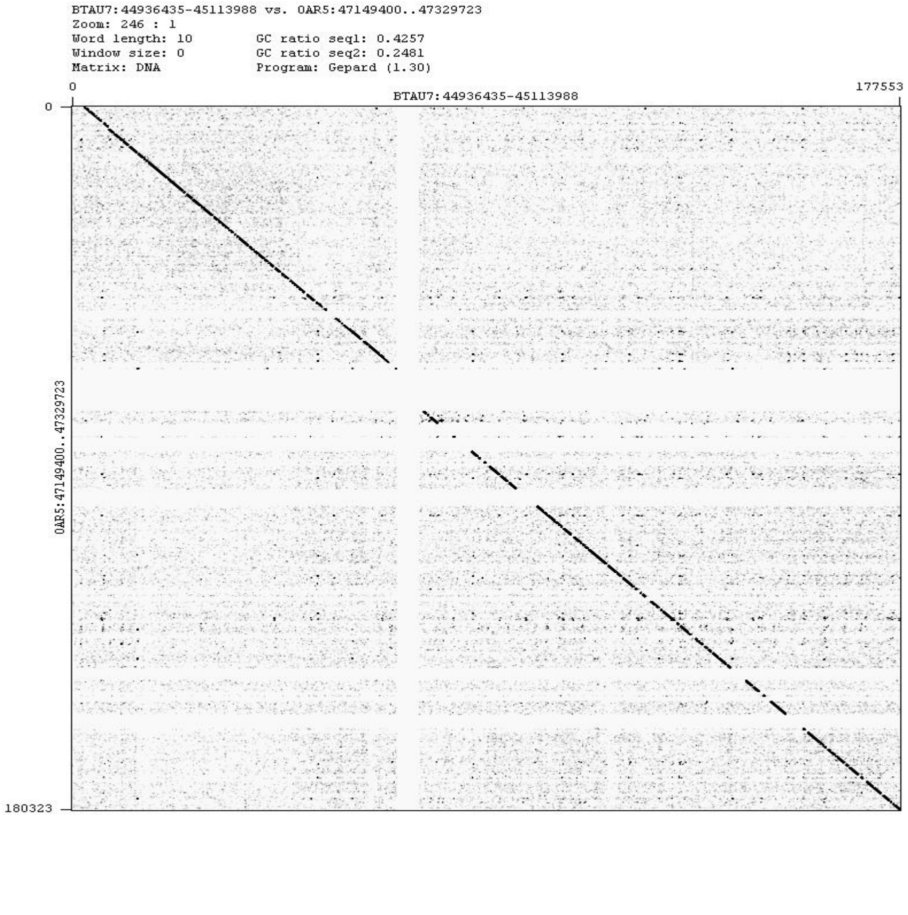
BTAU7:44936435-45113988 vs. OAR5:47149400..47329723
Zoom: 246 : 1
Word length: 10
Window size: 0
Matrix: DNA
GC ratio seq1: 0.4257
GC ratio seq2: 0.2481
Program: Gepard (1.30)
0
BTAU7:44936435-45113988
177553
0
OAR5:47149400..47329723
180323

Chr. 7-1

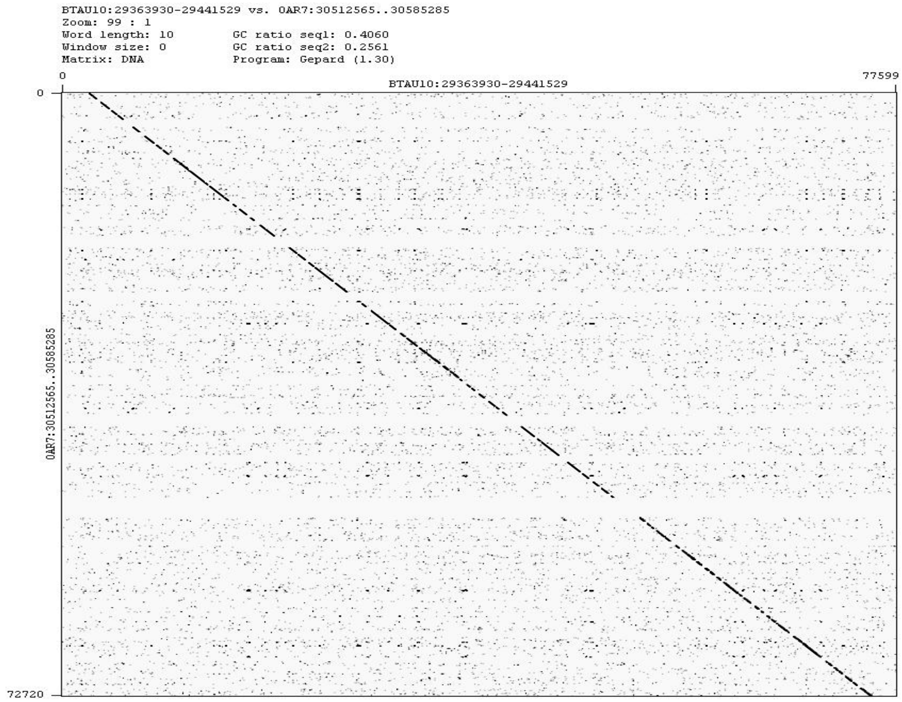
BTAU10:29363930-29441529 vs. OAR7:30512565..30585285
Zoom: 99 : 1
Word length: 10
GC ratio seq1: 0.4060
Window size: 0
GC ratio seq2: 0.2561
Matrix: DNA
Program: Gepard (1.30)
0
BTAU10:29363930-29441529
77599
0
OAR7:30512565..30585285
72720

Chr. 7-2

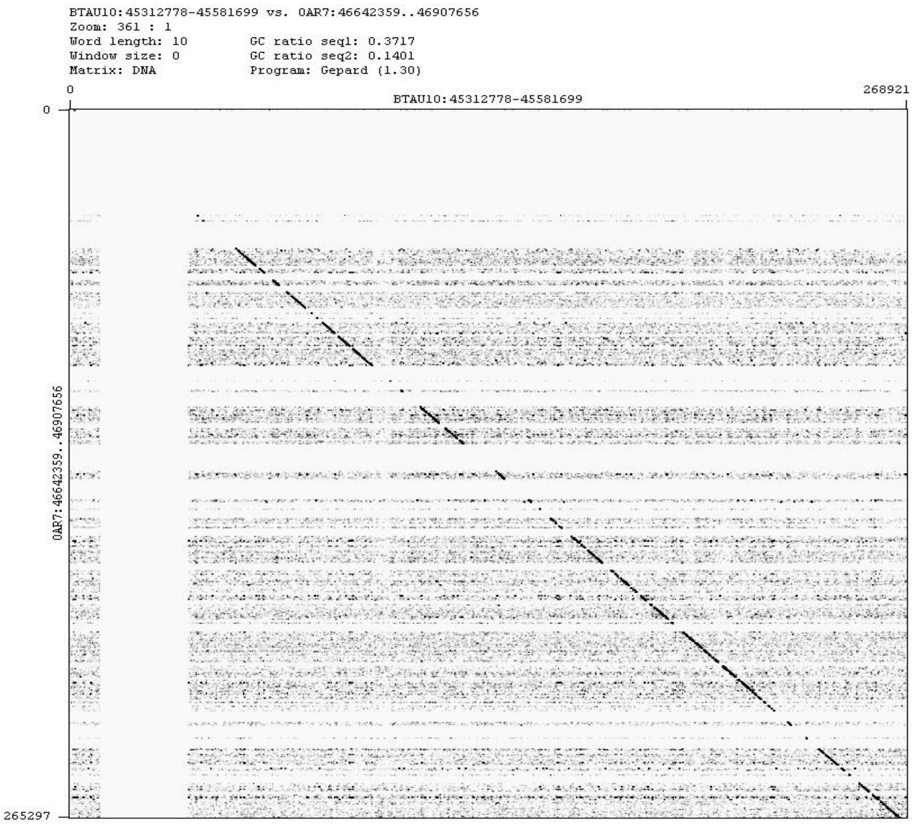
BTAU10:45312778-45581699 vs. OAR7:46642359..46907656
Zoom: 361 : 1
Word length: 10
GC ratio seq1: 0.3717
Window size: 0
GC ratio seq2: 0.1401
Matrix: DNA
Program: Gepard (1.30)
0
BTAU10:45312778-45581699
268921
0
OAR7:46642359..46907656
265297

Chr. X

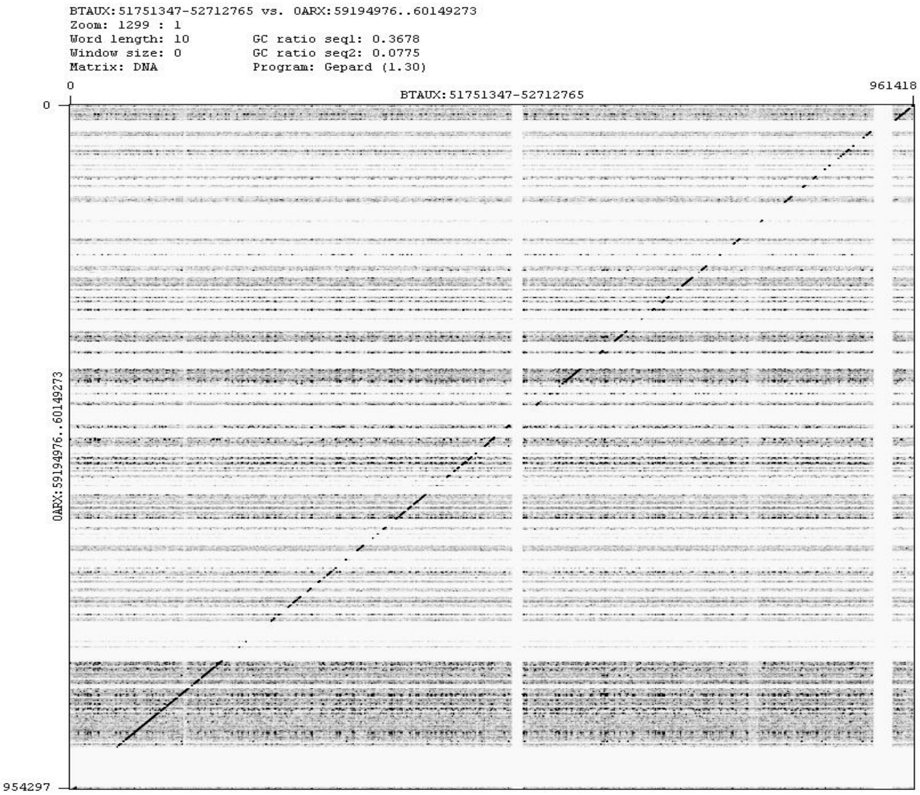
BTAUX:51751347-52712765 vs. OARX:59194976..60149273
Zoom: 1299 : 1
Word length: 10
Window size: 0
Matrix: DNA
GC ratio seq1: 0.3678
GC ratio seq2: 0.0775
Program: Gepard (1.30)
0
961418
BTAUX:51751347-52712765
0
OARX:59194976..60149273
954297
