# Supplementary material for: Genomic scan of selective sweeps in thin and fat tail sheep breeds for identifying of candidate regions associated with fat deposition
Source: BMC Genet. 2012 Feb 26;13:10. doi: 10.1186/1471-2156-13-10 (PMC3351017; doi:10.1186/1471-2156-13-10)

Additional file 2 – Change in effective population size across generations as estimated from linkage disequilibrium data.

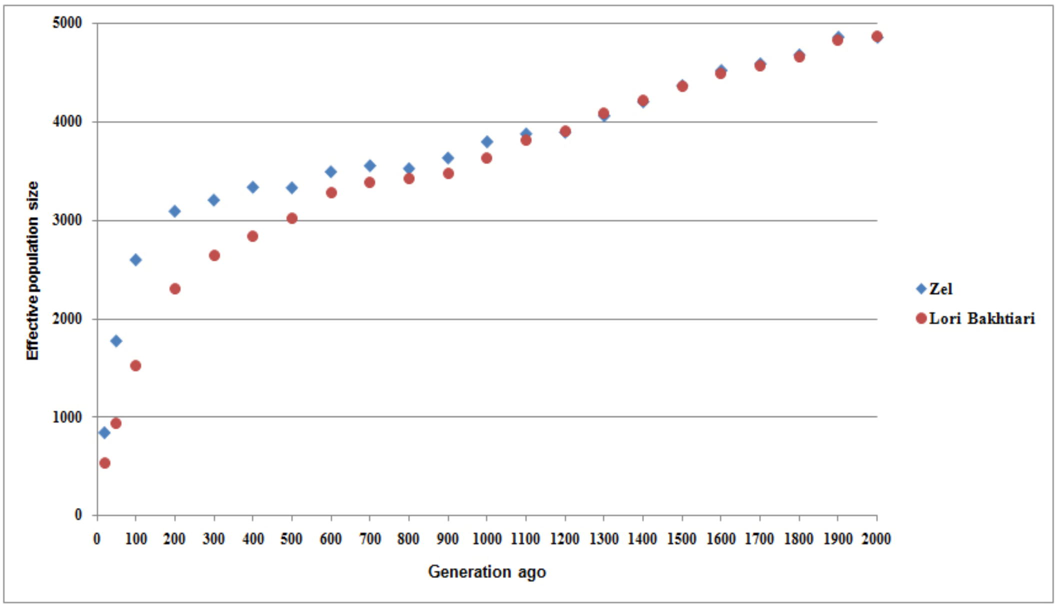

Supplement: Additional file 2 — Figure S2: Change in effective population size across generations as estimated from linkage disequilibrium data. [file 1471-2156-13-10-S2.PDF]
