# Supplementary material for: Genomic scan of selective sweeps in thin and fat tail sheep breeds for identifying of candidate regions associated with fat deposition
Source: BMC Genet. 2012 Feb 26;13:10. doi: 10.1186/1471-2156-13-10 (PMC3351017; doi:10.1186/1471-2156-13-10)
Supplement: Additional file 3 — Table S3: Summary table for data cleaning in the Zel-Lori Bakhtiari data set. [file 1471-2156-13-10-S3.PDF]

Additional file 3 – Summary table for data cleaning in Zel-Lori Bakhtiari data set

All SNPs (49018)	Zel (Thin tailed)	Lori Bakhtiari (Fat tailed)
Number of Animals	47	47
Excluding Animals >5% Genotype Failure	1	1
Excluding regard to PCA results	1	1
Remaining Animals	45	45
Number of SNPs	49018	49018
Excluding markers ≤2% MAF over all animals	1462	1462
SNPs remaining	47576	47576
Excluding SNPs <95% Call over all animals	1594	1594
SNPs remaining	45963	45963
Excluding SNPs with deviation from HW(<0.000001)	20	15
SNPs remaining	45928	45928
Excluding Unknown SNPs	317	317
SNPs remaining	45611	45611
