# Supplementary material for: Genomic scan of selective sweeps in thin and fat tail sheep breeds for identifying of candidate regions associated with fat deposition
Source: BMC Genet. 2012 Feb 26;13:10. doi: 10.1186/1471-2156-13-10 (PMC3351017; doi:10.1186/1471-2156-13-10)
Supplement: Additional file 4 — Table S4: Summary table for data cleaning in the Ovine HapMap data set. [file 1471-2156-13-10-S4.PDF]

Additional file 4 - Summary table for data cleaning in HapMap data set

All SNPs (49018)	Thin tailed	Fat tailed
Number of Animals	197	75
Excluding markers ≤2% MAF over all animals	331	331
SNPs remaining	48687	48687
Excluding SNPs <95% Call over all animals	310	310
SNPs remaining	48377	48377
Excluding Unknown SNPs	324	324
SNPs remaining	48053	48053
